# What stresses men? predictors of perceived stress in a population-based multi-ethnic cross sectional cohort

**DOI:** 10.1186/1471-2458-13-113

**Published:** 2013-02-06

**Authors:** Timothy R Rebbeck, Anita L Weber, Elaine Spangler, Charnita M Zeigler-Johnson

**Affiliations:** 1 Department of Biostatistics and Epidemiology, University of Pennsylvania School of Medicine, 217 Blockley Hall, 423 Guardian Drive, Philadelphia, PA, 19104-6021, USA; 2 Abramson Cancer Center, University of Pennsylvania School of Medicine, Philadelphia, PA, 19104, USA

**Keywords:** Neighborhoods, Ethnicity, Age

## Abstract

**Background:**

Perceived stress (PS) is a risk factor for a variety of diseases. However, relatively little is known about age- or ethnicity-specific differences in the effect of potential predictors of PS in men.

**Methods:**

We used a population-based survey of 6,773 White, 1,681 Black, and 617 Hispanic men in Southeastern Pennsylvania to evaluate the relationship of self-reported PS and financial security, health status, social factors, and health behaviors. Interactions across levels of age and ethnicity were tested using logistic regression models adjusted for overall health status, education, and household poverty.

**Results:**

High PS decreased significantly with age (p < 0.0001) and varied by ethnicity (p = 0.0001). Exposure to health-related and economic factors were more consistently associated with elevated PS in all ethnicities and ages, while social factors and health behaviors were less strongly or not at all associated with PS in most groups. Significant differences in the relationship of high PS by age and ethnicity were observed among men who are medically uninsured (p = 0.0002), reported missing a meal due to cost (p < 0.0001), or had spent a night in the hospital (p = 0.020). In contrast, not filling a prescription due to cost and diagnosed with a mental health condition were associated with high PS but did not differ by age and ethnicity subgroup.

**Conclusions:**

These data suggest that some, but not all, factors associated with high PS differ by age and/or ethnicity. Research, clinical, or public health initiatives that involve social stressors should consider differences by age and ethnicity.

## Background

There is substantial evidence from both animal and human studies that exposure to chronic and acute stress has negative health effects. These stressors include social relationships such as social isolation, exposures, lifestyle, socio-economic status, and many other classes of stressors. Selye [1] defined a variety of physiological responses to stressors that may affect human health. Acute adaptive responses to stressor exposure include energy mobilization (e.g., glucose mobilization, increased blood pressure and heart rate), cognitive and sensory changes, and a sharpening of memory. However, exposure to stressors also confers maladaptive responses including increased inflammatory processes, hypertension, reproductive abnormalities, changes to the immune system, and metabolic diseases. As reviewed by Antoni et al. [2], exposure to stressors leads to physiological reactivity at a variety of levels, including effects through endocrine (e.g., cortisol) also and sympathetic ganglia (e.g., noradrenaline) pathways. Studies have connected exposure to specific stressors with negative health consequences. For example, Berkman and Syme [3] reported that all-cause mortality was significantly higher in those individuals who were the least socially integrated compared to those with the highest level of social integration. Among women ages 50–59, the relative risk associated with social integration was doubled (RR = 2.1). In men ages 50–59, this effect was even greater (RR = 3.2). These and other data suggest that factors associated with stress have negative health consequences, and that some stressors may have more deleterious effects in men than in women. Despite this and other data, comprehensive studies of the effects of social factors on stress in men of different ethnicities or ages are limited.

To evaluate the role of disparities in social factors on perceived stress (PS) in adult men by ethnicity and age group, we evaluated the effect of potential correlates of PS by age and ethnicity in a large, population-based sample of men.

## Methods

The study sample for these analyses comes from the combined Community Health Data Bases (CHDB) of 2002, 2004 and 2006, a biannual random digit dial telephone survey of more than 10,000 households to examine the health and social well-being of residents in Bucks, Chester, Delaware, Montgomery, and Philadelphia counties in Southeastern Pennsylvania. The survey is conducted as part of Philadelphia Health Management Corporation’s (PHMC) CHDB, which contains information about local residents' health status, use of health services, and access to care. PHMC is a nonprofit, public health organization committed to improving the health of the community through outreach, education, research, planning, technical assistance, and direct services. The central component of the CHDB is the biannual Southeastern Pennsylvania Household Health Survey of residents in Southeastern Pennsylvania.

According to the definition provided by the CHDB, 269 neighborhoods were identified. Neighborhoods were consistent across these 3 waves of data with a couple of exceptions where suburban neighborhoods were subdivided from one unit into two across the waves. For the combined dataset, the two neighborhoods were recombined to the original neighborhood. The sample was stratified over 54 service areas with approximately 30,000 to 75,000 adult residents in each recruitment area. In 2006, older persons (60+) and Latinos were oversampled. In 2002 and in 2004, older persons (60+) and Asians were oversampled. The CHDB survey is designed to be representative of the 5-county region at the county and Zip Code levels. It is designed to be representative in terms of race, age, gender, poverty level, and household size (based on projections from the 2000 Census.) Weights ranging from 0.285 to 3.154 are available to adjust for sampling bias by giving adding weight to underrepresented segments of the population and decreased weight to overrepresented segments. However, previous analyses showed that the use of unweighted data did not substantially change results. For these analyses, we merged the 2002, 2004 and 2006 CHDB adult datasets, comprising roughly 30,000 individuals 18 years and older in the 269 neighborhoods. Respondents were ages 18–100 (mean age = 49 years.) The survey’s response rate was 32% in 2002, 27% in 2004, and 24% in 2006.

For the present study, the subset of N= 9,071 White, Black, or Hispanic (of any ethnicity) adult men were eligible from among the 30,548 men and women of all ethnicities aged 18 and over who completed the survey. Ethnicity was determined by participant self-identification using the CHDB questionnaire categories. All eligible men had a value for age, ethnicity, and self-reported stress, the outcome of interest. These men were distributed across 269 communities in the 5-county region with a slightly higher representation from the suburban counties (Bucks, Chester, and Montgomery) and a slightly lower representation from the urban counties (Philadelphia and Delaware which includes the city of Chester) than the sample as a whole.

These data were used to conduct cross-sectional analyses of individual-level stress score with potential individual-level stressors. Self-reported PS is a metric devised by the CHDB to assess an overall measure of PS in southeast Pennsylvanians. PS is assessed by a 10-point scale, where “1” meant “no stress” and “10” meant “an extreme amount of stress”, in answer to the question “How much stress would you say you have experienced during the past year?” The distribution of self-reported stress was roughly uniform. We dichotomized the self-reported stress to ‘0’ if the stress level was < 7 (low to moderate) and ‘1’ if the stress level was ≥ 7 (high) in the past year.

Age was evaluated by the fill-in question ‘What is your age please?’ Race/ethnicity was evaluated by two back-to-back questions: ‘Are you of Hispanic or Latino origin or descent?’ and ‘What ethnicity to you consider yourself to be?’ with nine response options: ‘White/Caucasian, Black/African American, Asian, Latino/Hispanic, Biracial/Mixed, Native American/American Indian, Other, Don’t Know, and Refused’. To be consistent with the question being asked here, we use the terms “White”, “Caucasian”, “Black”, “African American” and “Hispanic” throughout. Due to small numbers, other races were not considered in the analyses presented here.

A subset of the available variables was selected to represent a variety of potential PS correlates. These variables represent socio-economic status, financial security, health status, social factors, and lifestyle factors. First, we evaluated three factors in each of the models as potential confounder variables, which were recorded as answers to the following questions:

1. Overall Health: Would you say your health, in general, is excellent, good, fair, or poor? (Collapsed to 3 categories: excellent, good, and fair/poor).

2. Educational Attainment: What was the last grade of school that you completed? (Collapsed from 5 categories to less than high school graduate (0–11 years), high school graduate (12 years), and some college or more (more than 12 years of education).

3. Poverty: Living below 100%, at or above the 100% but below 150%, or at or above 150% of the federal poverty level was a computed variable based on family income and number of people living in the household or was imputed by the PHMC using other variables if family income and/or number of people living in the household were missing.

Second, we considered a series of potential stressors in broad categories that may be associated with PS, defined by the responses to a series of questions listed below. These include:

4. Financial Security:

a. Did you or other adults in your household ever cut the size of meals or skip meals in the past 12 months because there was not enough money in the budget for food?

b. Did not fill a prescription in the past year because of cost?

c. Currently have health insurance? If yes, have been without health insurance coverage at any time in the past year? (Combined to Yes/No medically uninsured currently or at any time in the past year?)

5. Health Status:

a. Have health problem or condition that requires medical treatment or hospitalization on a regular basis?

b. Have you ever been diagnosed with any mental health condition, including clinical depression, anxiety disorder or bipolar disorder?

c. How many times have you been a patient overnight in a hospital during the past 12 months? (Dichotomized to Yes/No In a hospital overnight during the past 12 months?)

6. Social Factors:

a. How many people, including yourself, are currently living in your household? (Dichotomized to Yes/No Living alone?)

b. Have you been subject to any kind of physical violence by friends, family members or strangers, such as being shoved, slapped, beaten, forced into sexual activity or threatened or hurt with a knife or gun?

c. Are there any firearms, such as handguns, shotguns, or rifles in or around your home?

7. Health Behaviors:

a. How many times per week did you participate in any physical activities for exercise that lasted for at least one-half hour in the past month? (Four response categories dichotomized to Yes/No more than 3 days/week?)

b. Have you smoked at least 100 cigarettes in your entire life? Do you now smoke cigarettes every day, some days, or not at all? (Combined to never, current, or former smoker?)

## Statistical methods

Estimates of association between potential stressors with 1:Yes, 0:No high perceived stress (PS) were obtained using multivariate logistic regression models. In the first series of models, high PS was modeled with a base model of the main effect of ethnicity in 3 categories (White, Black, Hispanic of any ethnicity) and the main effect of age in 4 categories (18–39, 40–54, 55–64, 65+). To this base model, each factor was added one at a time to evaluate its main effect. In the second series of models, high PS was modeled by the three 2-way interactions (factor by ethnicity, factor by age and ethnicity by age) and the nested main effects of factor, ethnicity, and age. In the third and last series, high PS was modeled by the single 3-way interaction (factor by ethnicity by age) and the nested 2-way interactions and main effects in order to assess the significance of adding the 3-way interaction above modeling with the two-way interactions alone. To estimate the significance of adding a factor as a main effect and interaction terms, nested models were compared using the likelihood ratio test. All models described above were adjusted for potential confounding with overall health, educational attainment, and household poverty level as 3-level categorical variables as described in the Methods. In order to test the effects of each potential stressors across the age- and ethnicity-specific strata, we identified a common reference group (i.e., White men age 65+). This choice of reference group allowed us to provide a consistent comparison of the effects of age and ethnicity for all variables. The odds ratios (ORs) and 95% confidence intervals (CIs) for each factor at each ethnicity-age group in the third series were estimated with white men aged 65 years or more with the lowest value of the factor (typically ‘0:No’) as the reference category. These ORs were graphed for a more intuitive presentation of potential effect modification of ethnicity and age on the association of the factor with high PS. Individuals with missing data values were not excluded from the data set. Instead, those individuals were removed from the specific analyses in which the variable was included. Two-sided p-values <0.05 were considered significant. All analyses, including graphs, were performed in STATA (version 10.1, STATA Corporation, College Station, TX).

## Results

Table 1 presents the distribution of high PS by age and ethnicity. High PS was reported most commonly at the youngest ages and generally decreased with each age interval in each ethnicity (p < 0.0001 overall and p < 0.0001 among Whites, p < 0.0001 among Blacks and p = 0.003 among Hispanics) in adjusted models. Unadjusted associations were similar. High PS was most common among Whites, with Blacks and Hispanics nearly the same, after adjustment (p = 0.0001). In unadjusted models, the proportion of men reporting high PS was similar in all ethnicities (p = 0.17).

**Table 1:** Perceived Stress (PS) by ethnicity and age group, sample size and percentage

**Ethnicity**	**White, N (%)**					**Black, N (%)**					**Hispanic, N (%)**				
Age	18-39	40-54	55-64	65+	Total	18-39	40-54	55-64	65+	Total	18-39	40-54	55-64	65+	Total
N	1876	2164	1211	1522	6773	584	513	262	322	1681	327	180	66	44	617
High PS	667 (36)	717 (33)	276 (23)	172 (11)	1832 (27)	197 (34)	147 (29)	54 (21)	43 (13)	441 (26)	109 (33)	51 (28)	22 (33)	4 (9)	186 (30)
Low to Moderate PS	1209 (64)	1447 (67)	935 (77)	1350 (89)	4941 (73)	387 (66)	366 (71)	208 (79)	279 (87)	1240 (74)	218 (67)	129 (72)	44 (67)	40 (91)	431 (70)

Potential correlates of PS include those associated with financial security, health status, social factors, and health behaviors. The distribution of these variables by ethnicity and age is presented in Additional file 1: Table S1. For each potential stressor, we report the p-values and degrees of freedom of the main effect overall and stratum-specific effects by age and/or ethnicity.

**Figure 1:**
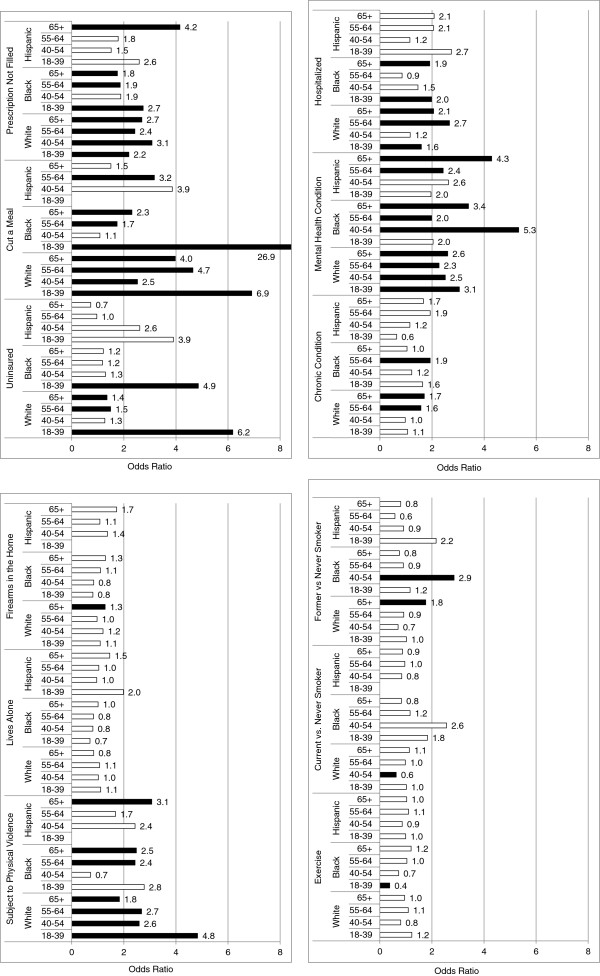
**Main effects of putative stressors and perceived stress, by age and ethnicity, adjusted for poverty, education, and overall health.** Groups without values indicate no odds ratio could be estimated

As expected, many of the stressors studied here were individually associated with PS, although there was substantial heterogeneity in these estimates by age and ethnicity. The main effect of each stressor is presented in Figure 1 and Additional file 2: Table S2. The PS of White men of all ages was affected by economic indicators including being uninsured, having cut a meal, not filled a prescription. These variables were only significant stressors in Black men over age 55 and in Hispanics ages 55–64. Health-related stressors including having a mental health condition or being hospitalized (except ages 40–54) were also associated with PS in White men of all ages. White men age 55+ experienced significantly increased PS associated with having a chronic condition. These variables were only associated with older age groups in Black and Hispanic men. An exception was the association of being hospitalized and an association with PS in younger (18–39 year old) Black men. Finally, social factors such as living alone (except Whites over age 65), exercise, and smoking were not significantly associated with PS in any age or ethnic group.

**Figure 2:**
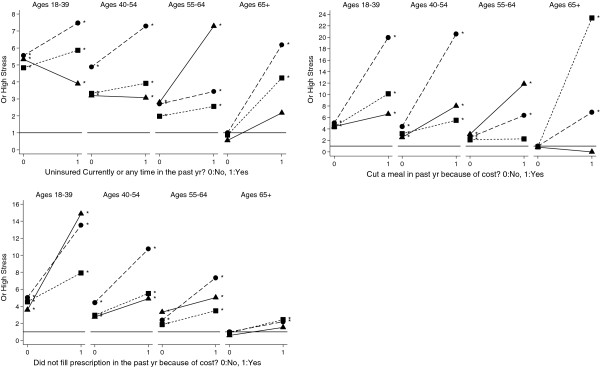
**Adjusted odds ratio effects of potential stressors, age and ethnicity: Financial security.** The reference category (OR = 1) is the oldest white men without the stressor. Models are adjusted by overall health, educational attainment, and household poverty. Asterisk indicates OR is significantly different from 1.0 reference category at the p<.05 level. Symbols denote ethnicity: ● White, ■ Black, ▲ Hispanic

**Figure 3:**
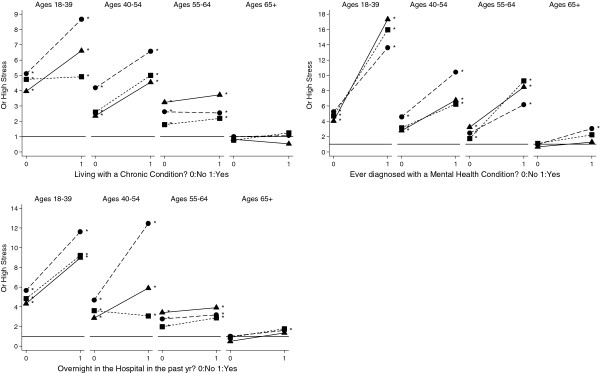
**Adjusted odds ratio effects of potential stressors, age and ethnicity: Health status.** The reference category (OR=1) is the oldest white men without the stressor. Models are adjusted by overall health, educational attainment, and household poverty. Asterisk indicates OR is significantly different from 1.0 reference category at the p<.05 level. Symbols denote ethnicity: ● White, ■ Black, ▲ Hispanic

**Figure 4:**
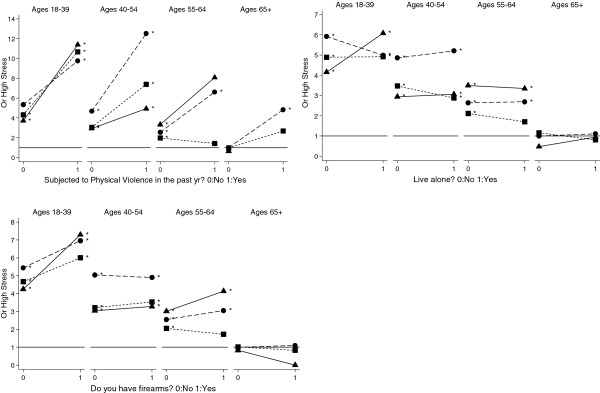
**Adjusted odds ratio effects of potential stressors, age and ethnicity: Social factors.** The reference category (OR=1) is the oldest white men without the stressor. Models are adjusted by overall health, educational attainment, and household poverty. Asterisk indicates OR is significantly different from 1.0 reference category at the p<.05 level. Symbols denote ethnicity: ● White, ■ Black, ▲ Hispanic

**Figure 5:**
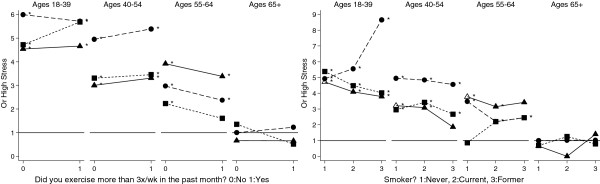
**Adjusted odds ratio effects of potential stressors, age and ethnicity: Health behavior.** The reference category (OR=1) is the oldest white men without the stressor. Models are adjusted by overall health, educational attainment, and household poverty. Asterisk indicates OR is significantly different from 1.0 reference category at the p<.05 level. Symbols denote ethnicity: ● White, ■ Black, ▲ Hispanic

To more formally quantify differences in the relationships of putative stressors on PS by age and ethnicity, we present the odds ratios (OR) comparing each ethnicity and age group against a common reference group (i.e., White men age 65 and over with the lowest value of the factor; Figures 2, 3, 4, 5 and Additional file 3: Table S3). In general, and as denoted by an asterisk at each point in Figures 2, 3, 4, 5, most age- and ethnicity-specific groups were associated with increased odds of reporting high PS compared with the reference group. In many cases, the OR estimates were very high among those reporting the stressor, with estimates greater than OR = 5 in many strata. The main exception is within the oldest age group (65+), where there was only limited evidence for associations of stressors with high PS.

With respect to financial security (Figure 2), there were significant differences in the effect of being medically uninsured or having cut a meal in the past year due to cost by age and ethnicity (p < 0.001 in all tests of interaction, Table 2). Being medically uninsured was most strongly associated with high PS among White men at all ages, and among older middle age (55–64) Hispanic men and older (65+) Black men. Having cut a meal due to cost was most strongly associated with high PS in younger (18–39 and 40–54) White men, middle age (55–64) Hispanic men, and older (65+) Black men. There was no significant difference in the association of having not filled a prescription by age and ethnicity (p > = 0.244, Table 2) and as shown in Figure 2 by approximately parallel lines.

**Table 2:** Test of interaction of stress covariates with perceived stress (PS) by age and ethnicity, p-value and degrees of freedom

		**H**_**A**_**:**	Three-Way Interactions*	Two-Way Interactions* +Main Effects with Factor	**Main Effects Only with Factor**
			+ Two-Way Interactions*		
			**+ Main Effects with Factor**		
		**H**_**o**_**:**	**Main Effects Only with Factor**	**Main Effects Only with Factor**	**Main Effects Only without Factor**
**Category**	**Factor**	**Levels**			
Confounders	Overall Health	Excellent, Good, Fair/Poor	--	--	p<0.0001, df=2
	Education in years	< 12, 12, > 12	--	--	p<0.0001, df=2
	Household Proverty as% of Poverty Threshold	> = 150%, 100 = < 150%, < 100%	--	--	p<0.0001, df=2
Financial Security	Medically Uninsured (past yr)	Yes, No	p=0.0002, df=17	p<0.0001, df=11	p<0.0001, df=1
	Missed a Meal due to Cost (past year)	Yes, No	p<0.0001, df=16^†^	p=0.0002, df=11	p<0.0001, df=1
	Did not Fill a Prescription due to Cost (past year)	Yes, No	p=0.343, df=17	p=0.244, df=11	p<0.0001, df=1
Health Status	Living with a Chronic Condition	Yes, No	p=0.058, df=17	p=0.023, df=11	p<0.0001, df=1
	Ever Diagnosed with a Mental Health Condition	Yes, No	p=0.364, df=17	p=0.190, df=11	p<0.0001, df=1
	Overnight in Hospital (past year)	Yes, No	p=0.020, df=17	p=0.052, df=11	p<0.0001, df=1
Social Factors	Subject to Physical Violence (past year)	Yes, No	p=0.503, df=16^†^	p=0.378, df=11	p<0.0001, df=1
	Live Alone	Yes, No	p=0.517, df=17	p=0.337, df=11	p=0.668, df=1
	Firearms in Home	Yes, No	p=0.582, df=16^†^	p=0.300, df=11	p=0.047, df=1
Health Behaviors	Exercise more than 3x per Week (past month)	Yes, No	p=0.130, df=17	p=0.202, df=11	p=0.905, df=1
	Smoker	Never, Current, Former	p=0.054, df=27	p=0.130, df=16	p=0.896, df=2

*Three-way interaction: factor by age by Ethnicity; Two-way interactions: factor by age, factor by Ethnicity, age by Ethnicity.

^†^ Hispanic-Oldest age (65+) subgroup was dropped from the model because only “No High PS” outcome was observed.

As shown in Table 2, some associations between health status and high PS differed significantly by age and ethnicity. Interactions across age and ethnicity predicting high PS were evident for men who spent at least one overnight in the hospital (p = 0.020). As shown in Figure 3, the effect of overnight hospitalization was strongest in younger men of all ethnicities, and in middle age (40–54) White men. Our results also show that high PS is associated with an interaction of living with a chronic condition with age (p < 0.009) but not with ethnicity (p = 0.762). While the combination of all three 2-way interactions, including age by ethnicity, is significant (p = 0.023) the 3-way interaction across levels of age and ethnicity is not. The effect of ever having been diagnosed with a mental health condition was highly significant as a main effect (p<0.0001) but did not differ significantly by ethnicity or age.

While the social factors (Figure 4) of subject to physical violence and firearms in the home were associated as main effects with increases in high PS, there was no evidence that the effect of these potential stressors differed significantly by age or ethnicity (Table 2). Health behaviors (Figure 5) showed neither significant main effects nor interactions with age and ethnicity (Table 2).

## Discussion

Our data suggest that there is substantial heterogeneity in the effect of the stressors studied here on high perceived stress (PS) by ethnicity and age. Our results suggest that the overall trend indicates PS diminishes with age for most putative stressors, but that there are substantial age- and ethnicity-specific differences in these patterns. In general, the factors studied here were more strongly associated with PS in White men, and least associated with PS in Hispanic men. These results suggest that interventions that attempt to mediate stress or that study these stressors as disease risk factors should be aware the age and ethnicity modulate some (but not all) of these relationships.

We have made a number of assumptions in the model choices we have made that could have influenced our results. We undertook logistic models using a common reference group to evaluate the association with PS of each level of stressor by age and ethnicity. The relationships among these variables are likely to be complex, and the assumption of log-linearity applied here may not be appropriate. While this study was a population-based survey, there is the potential that the sampling design or the low response rate led to biases in our analyses. While our overall sample was relatively large, the subgroup of Hispanic men was substantially smaller and it is possible we were not able to detect interactions involving Hispanic men due to sample size limitations. In addition, the response rate in some groups was low (e.g., < 50% of all eligible respondents). Therefore, the generalizability of the results may be limited, and not represent the general populations from which this sample was drawn. Despite the large size of some groups, some odds ratio estimates were generated from smaller sample strata, which resulted in large confidence intervals and less stable inferences. For these groups, caution should be used when interpreting the data. Finally, we adjusted for stressors that reflect overall health status, education, and household poverty to correct for differences among strata that may lead to confounding. However, other confounders may be acting that were not measured in this study, and therefore we cannot rule out the potential that some residual confounding remains in this analysis.

Despite these limitations, there is a large literature that supports the hypothesis that stressors have an important impact on health status, and therefore differences in the relationship of these factors by age or ethnicity could (in part) explain some health disparities. It is clear from both animal and human studies that exposure to chronic stress and an individual’s behavioral or physiological response to those stressors is relevant to their health. In men, exposure to stressors has a variety of biological effects that have an influence on health, including vulnerability to brain-dependent and stress-related mental and physical health conditions [4], susceptibility to frailty in aging adults [5], reproductive impairment [6,7], obesity and metabolic syndrome [8], cardiovascular stress reactivity [9], and a variety of biomarkers of endocrine dysfunction, inflammation or allostatic load that are correlated with altered health and cognition [10-12]. In addition, therapeutic interventions or altering social interactions to reduce or manage stress are available [13-16]. However, the literature in these domains has generally not explored the potential that these relationships differ significantly by age or ethnicity. Our results suggest that the stressors studied here are more likely to be associated with PS in White men compared with Hispanic men. This may mean that the stressors measured here are more relevant to White men and other factors may be more relevant to Hispanic men. However, if our data are confirmed and extended to other phenotypes, the information provided here may assist in developing tailored studies or strategies for the reduction of stress-related health disparities.

## Conclusions

Our results suggest that factors associated with self-reported perceived stress vary by age and ethnicity, with perceived stress diminishing with age. Public health and other approaches that wish to intervene on perceived stress and its disease correlates should account for age- and ethnic-specific differences.

## Competing interests

The authors have no financial or non-financial competing interests to declare.

## Authors’ contributions

TR, ES and CZ were responsible for the generation and processing of the data used in this analysis. AW carried out the data analysis. TR and CZ conceived of the study, and participated in its design and coordination. All authors were involved directly in drafting portions of the manuscript. All authors read and approved the final manuscript.

## Pre-publication history

The pre-publication history for this paper can be accessed here:

http://www.biomedcentral.com/1471-2458/13/113/prepub

## Supplementary Material

Additional file 1: Table S1 Covariate Distribution by Ethnicity and Age Group, sample size and percentage. Click here for file

Additional file 2: Table S2 Main Effects of Each Stressor on Perceived Stress, adjusted for Overall Health, Education, and Income. Click here for file

Additional file 3: Table S3 OR Estimates with Lower and Upper Bound of the 95% C. Click here for file
