# Supplementary material for: What stresses men? predictors of perceived stress in a population-based multi-ethnic cross sectional cohort
Source: BMC Public Health. 2013 Feb 6;13:113. doi: 10.1186/1471-2458-13-113 (PMC3627635; doi:10.1186/1471-2458-13-113)
Supplement: Additional file 1: Table S1 — Covariate Distribution by Ethnicity and Age Group, sample size and percentage. [file 1471-2458-13-113-S1.doc]

**Supplementary Table 1. Covariate Distribution by Ethnicity and Age Group, sample size and percentage**

|  | Ethnicity | White, N (%) | | | | |  | Black, N (%) | | | | |  | Hispanic, N (%) | | | | |
| --- | --- | --- | --- | --- | --- | --- | --- | --- | --- | --- | --- | --- | --- | --- | --- | --- | --- | --- |
|  | Age | 18-39 | 40-54 | 55-64 | 65+ | Total |  | 18-39 | 40-54 | 55-64 | 65+ | Total |  | 18-39 | 40-54 | 55-64 | 65+ | Total |
|  | Variable | N=1876 | N=2164 | N=1211 | N=1522 | N=6773 |  | N=584 | N=513 | N=262 | N=322 | N=1681 |  | N=327 | N=180 | N=66 | N=44 | N=617 |
| Covariates Included in all Models | Overall Health | | | | | |  |  |  |  |  |  |  |  |  |  |  |  |
|  | Excellent | 879(47) | 853(39) | 409(34) | 296 (19) | 2437(36) |  | 220(38) | 109(21) | 37(14) | 41(13) | 407(24) |  | 108(33) | 51(28) | 12(18) | 11(25) | 182(30) |
|  | Good | 851(45) | 1052(49) | 541(45) | 789(52) | 3233(48) |  | 296(51) | 248(48) | 124(47) | 118(37) | 786(47) |  | 159(49) | 71(39) | 24(36) | 12(27) | 266(43) |
|  | Fair/Poor | 146(8) | 259(12) | 261(22) | 437(29) | 1103(16) |  | 68(12) | 156(30) | 101(39) | 163(51) | 488(29) |  | 60(18) | 58(32) | 30(45) | 21(48) | 169(27) |
|  | Educational Attainment | | | | | |  |  |  |  |  |  |  |  |  |  |  |  |
|  | Less Than High School Graduate | 77 (4) | 61 (3) | 68 (6) | 192(13) | 398(6) |  | 52(9) | 55(11) | 47(18) | 123(38) | 277(16) |  | 75(23) | 42(23) | 22(33) | 20(45) | 159(26) |
|  | High School Graduate | 430(23) | 613(28) | 335(28) | 478(31) | 1856(27) |  | 220(38) | 203(40) | 116(44) | 106(33) | 645(38) |  | 119(36) | 49(27) | 25(38) | 13(30) | 206(33) |
|  | Some College or more | 1369(73) | 1490(69) | 808(67) | 852(56) | 4519(67) |  | 312(53) | 255(50) | 99(38) | 93(29) | 759(45) |  | 133(41) | 89(49) | 19(29) | 11(25) | 252(41) |
|  | Household Poverty (% of Household Poverty Threshold) | | | | | |  |  |  |  |  |  |  |  |  |  |  |  |
|  | High (>150%) | 1774(95) | 2038(94) | 1126(93) | 1356(89) | 6294(93) |  | 465(80) | 403(79) | 198(76) | 205(64) | 1271(76) |  | 228(70) | 121(67) | 41(62) | 26(59) | 416(67) |
|  | Medium (100%-<150%) | 60 (3) | 70 (3) | 48 (4) | 112(7) | 290(4) |  | 65(11) | 54(11) | 38(14) | 65(20) | 222(13) |  | 42(13) | 32(18) | 13(20) | 9(20) | 96(16) |
|  | Low (<100%) | 42 (2) | 56 (3) | 37 (3) | 54 (4) | 189(3) |  | 54(9) | 56(11) | 26(10) | 52(16) | 188(11) |  | 57(17) | 27(15) | 12(18) | 9(20) | 105(17) |
| Financial Security | Medically uninsured now or in the past year? | | | | | |  |  |  |  |  |  |  |  |  |  |  |  |
|  | Yes | 331(18) | 215(10) | 79 (7) | 39 (3) | 664(10) |  | 190(33) | 106(21) | 45(17) | 23(7) | 364(22) |  | 147(45) | 54(30) | 17(26) | 5(11) | 223(36) |
|  | No | 1543(82) | 1949(90) | 1131(93) | 1482(97) | 6105(90) |  | 393(67) | 406(79) | 217(83) | 299(93) | 1315(78) |  | 180(55) | 126(70) | 49(74) | 39(89) | 394(64) |
|  | Cut a meal in past 12 months because of cost? | | | | | |  |  |  |  |  |  |  |  |  |  |  |  |
|  | Yes | 129(7) | 124(6) | 49 (4) | 26 (2) | 328(5) |  | 85(15) | 69(13) | 19(7) | 13(4) | 186(11) |  | 45(14) | 31(17) | 10(15) | 4(9) | 90(15) |
|  | No | 1743(93) | 2039(94) | 1161(96) | 1495(98) | 6438(95) |  | 499(85) | 442(86) | 242(92) | 309(96) | 1492(89) |  | 281(86) | 149(83) | 56(85) | 40(91) | 526(85) |

|  | Did not fill prescription in past yr because of cost? | | | | | |  |  |  |  |  |  |  |  |  |  |  |  |
| --- | --- | --- | --- | --- | --- | --- | --- | --- | --- | --- | --- | --- | --- | --- | --- | --- | --- | --- |
|  | Yes | 176(9) | 197(9) | 88 (7) | 86 (6) | 547(8) |  | 83(14) | 98(19) | 35(13) | 41(13) | 257(15) |  | 46(14) | 34(19) | 16(24) | 5(11) | 101(16) |
|  | No | 1698(91) | 1966(91) | 1122(93) | 1433(94) | 6219(92) |  | 501(86) | 413(81) | 227(87) | 280(87) | 1421(85) |  | 279(85) | 146(81) | 50(76) | 39(89) | 514(83) |
