# Supplementary material for: What stresses men? predictors of perceived stress in a population-based multi-ethnic cross sectional cohort
Source: BMC Public Health. 2013 Feb 6;13:113. doi: 10.1186/1471-2458-13-113 (PMC3627635; doi:10.1186/1471-2458-13-113)
Supplement: Additional file 2: Table S2 — Main Effects of Each Stressor on Perceived Stress, adjusted for Overall Health, Education, and Income. [file 1471-2458-13-113-S2.doc]

**Supplementary Table 2: Main Effects of Each Stressor on Perceived Stress, adjusted for Overall Health, Education, and Income.**

| **Variable** | **Ethnicity** | **Age Group** | **OR** | **95%CI Lower Bound** | **95%CI Upper Bound** |
| --- | --- | --- | --- | --- | --- |
| Uninsured | Caucasian | 18-39 | 6.18 | 3.17 | 12.05 |
|  | Caucasian | 40-54 | 1.27 | 0.76 | 2.12 |
|  | Caucasian | 55-64 | 1.49 | 1.11 | 2.00 |
|  | Caucasian | 65+ | 1.35 | 1.05 | 1.72 |
|  | African American | 18-39 | 4.86 | 1.95 | 12.11 |
|  | African American | 40-54 | 1.29 | 0.61 | 2.74 |
|  | African American | 55-64 | 1.18 | 0.74 | 1.89 |
|  | African American | 65+ | 1.21 | 0.84 | 1.75 |
|  | Hispanic | 18-39 | 3.90 | 0.31 | 48.82 |
|  | Hispanic | 40-54 | 2.61 | 0.81 | 8.35 |
|  | Hispanic | 55-64 | 0.96 | 0.47 | 1.97 |
|  | Hispanic | 65+ | 0.73 | 0.46 | 1.18 |
| Cut a Meal | Caucasian | 18-39 | 6.91 | 3.10 | 15.43 |
|  | Caucasian | 40-54 | 2.52 | 1.40 | 4.56 |
|  | Caucasian | 55-64 | 4.65 | 3.08 | 7.02 |
|  | Caucasian | 65+ | 3.97 | 2.68 | 5.87 |
|  | African American | 18-39 | 26.88 | 6.93 | 104.20 |
|  | African American | 40-54 | 1.08 | 0.37 | 3.16 |
|  | African American | 55-64 | 1.74 | 1.02 | 2.96 |
|  | African American | 65+ | 2.31 | 1.43 | 3.72 |
|  | Hispanic | 18-39 |  |  |  |
|  | Hispanic | 40-54 | 3.86 | 0.94 | 15.76 |
|  | Hispanic | 55-64 | 3.17 | 1.39 | 7.19 |
|  | Hispanic | 65+ | 1.50 | 0.78 | 2.87 |
| Prescription not Filled | Caucasian | 18-39 | 2.19 | 1.29 | 3.75 |
|  | Caucasian | 40-54 | 3.08 | 1.96 | 4.84 |
|  | Caucasian | 55-64 | 2.42 | 1.78 | 3.28 |
|  | Caucasian | 65+ | 2.69 | 1.94 | 3.71 |
|  | African American | 18-39 | 2.74 | 1.26 | 5.96 |
|  | African American | 40-54 | 1.88 | 0.86 | 4.11 |
|  | African American | 55-64 | 1.86 | 1.17 | 2.97 |
|  | African American | 65+ | 1.75 | 1.08 | 2.81 |
|  | Hispanic | 18-39 | 2.59 | 0.21 | 31.87 |
|  | Hispanic | 40-54 | 1.51 | 0.47 | 4.89 |
|  | Hispanic | 55-64 | 1.78 | 0.81 | 3.92 |
|  | Hispanic | 65+ | 4.15 | 2.14 | 8.04 |
| Chronic Condition | Caucasian | 18-39 | 1.06 | 0.77 | 1.47 |
|  | Caucasian | 40-54 | 0.97 | 0.73 | 1.29 |
|  | Caucasian | 55-64 | 1.57 | 1.27 | 1.94 |
|  | Caucasian | 65+ | 1.70 | 1.24 | 2.33 |
|  | African American | 18-39 | 1.64 | 0.84 | 3.20 |
|  | African American | 40-54 | 1.22 | 0.66 | 2.26 |
|  | African American | 55-64 | 1.93 | 1.26 | 2.95 |
|  | African American | 65+ | 1.04 | 0.59 | 1.82 |
|  | Hispanic | 18-39 | 0.64 | 0.08 | 5.04 |
|  | Hispanic | 40-54 | 1.15 | 0.40 | 3.29 |
|  | Hispanic | 55-64 | 1.93 | 0.95 | 3.91 |
|  | Hispanic | 65+ | 1.67 | 0.85 | 3.27 |
| Mental Health Condition | Caucasian | 18-39 | 3.05 | 1.89 | 4.93 |
|  | Caucasian | 40-54 | 2.51 | 1.72 | 3.67 |
|  | Caucasian | 55-64 | 2.27 | 1.72 | 2.99 |
|  | Caucasian | 65+ | 2.60 | 1.92 | 3.51 |
|  | African American | 18-39 | 2.04 | 0.51 | 8.05 |
|  | African American | 40-54 | 5.33 | 1.99 | 14.22 |
|  | African American | 55-64 | 1.98 | 1.17 | 3.34 |
|  | African American | 65+ | 3.40 | 1.62 | 7.13 |
|  | Hispanic | 18-39 | 1.96 | 0.17 | 23.06 |
|  | Hispanic | 40-54 | 2.63 | 0.61 | 11.35 |
|  | Hispanic | 55-64 | 2.43 | 1.04 | 5.67 |
|  | Hispanic | 65+ | 4.29 | 1.97 | 9.36 |
| Hospitalized | Caucasian | 18-39 | 1.59 | 1.11 | 2.29 |
|  | Caucasian | 40-54 | 1.16 | 0.80 | 1.67 |
|  | Caucasian | 55-64 | 2.67 | 1.96 | 3.62 |
|  | Caucasian | 65+ | 2.06 | 1.37 | 3.09 |
|  | African American | 18-39 | 1.98 | 1.00 | 3.92 |
|  | African American | 40-54 | 1.46 | 0.71 | 2.98 |
|  | African American | 55-64 | 0.85 | 0.50 | 1.45 |
|  | African American | 65+ | 1.91 | 1.10 | 3.33 |
|  | Hispanic | 18-39 | 2.74 | 0.33 | 22.69 |
|  | Hispanic | 40-54 | 1.15 | 0.31 | 4.21 |
|  | Hispanic | 55-64 | 2.06 | 0.85 | 4.99 |
|  | Hispanic | 65+ | 2.08 | 0.96 | 4.48 |
| Subject to Physical Violence | Caucasian | 18-39 | 4.83 | 1.67 | 13.96 |
|  | Caucasian | 40-54 | 2.59 | 1.13 | 5.96 |
|  | Caucasian | 55-64 | 2.68 | 1.62 | 4.41 |
|  | Caucasian | 65+ | 1.83 | 1.27 | 2.63 |
|  | African American | 18-39 | 2.78 | 0.51 | 15.04 |
|  | African American | 40-54 | 0.72 | 0.08 | 6.42 |
|  | African American | 55-64 | 2.43 | 1.14 | 5.19 |
|  | African American | 65+ | 2.48 | 1.47 | 4.17 |
|  | Hispanic | 18-39 |  |  |  |
|  | Hispanic | 40-54 | 2.43 | 0.20 | 28.88 |
|  | Hispanic | 55-64 | 1.68 | 0.36 | 7.96 |
|  | Hispanic | 65+ | 3.07 | 1.58 | 5.96 |
| Lives Alone | Caucasian | 18-39 | 1.11 | 0.79 | 1.55 |
|  | Caucasian | 40-54 | 1.02 | 0.74 | 1.40 |
|  | Caucasian | 55-64 | 1.07 | 0.86 | 1.34 |
|  | Caucasian | 65+ | 0.84 | 0.66 | 1.07 |
|  | African American | 18-39 | 0.70 | 0.36 | 1.36 |
|  | African American | 40-54 | 0.81 | 0.42 | 1.53 |
|  | African American | 55-64 | 0.83 | 0.54 | 1.27 |
|  | African American | 65+ | 1.01 | 0.66 | 1.54 |
|  | Hispanic | 18-39 | 1.98 | 0.24 | 16.07 |
|  | Hispanic | 40-54 | 0.96 | 0.29 | 3.19 |
|  | Hispanic | 55-64 | 1.04 | 0.47 | 2.32 |
|  | Hispanic | 65+ | 1.46 | 0.76 | 2.81 |
| Firearms in the Home | Caucasian | 18-39 | 1.09 | 0.76 | 1.58 |
|  | Caucasian | 40-54 | 1.20 | 0.89 | 1.61 |
|  | Caucasian | 55-64 | 0.97 | 0.79 | 1.20 |
|  | Caucasian | 65+ | 1.28 | 1.02 | 1.61 |
|  | African American | 18-39 | 0.81 | 0.34 | 1.94 |
|  | African American | 40-54 | 0.84 | 0.40 | 1.77 |
|  | African American | 55-64 | 1.10 | 0.64 | 1.89 |
|  | African American | 65+ | 1.29 | 0.82 | 2.02 |
|  | Hispanic | 18-39 |  |  |  |
|  | Hispanic | 40-54 | 1.37 | 0.36 | 5.26 |
|  | Hispanic | 55-64 | 1.08 | 0.44 | 2.65 |
|  | Hispanic | 65+ | 1.72 | 0.85 | 3.49 |
| Exercise | Caucasian | 18-39 | 1.23 | 0.89 | 1.70 |
|  | Caucasian | 40-54 | 0.80 | 0.60 | 1.07 |
|  | Caucasian | 55-64 | 1.09 | 0.90 | 1.32 |
|  | Caucasian | 65+ | 0.95 | 0.78 | 1.16 |
|  | African American | 18-39 | 0.38 | 0.17 | 0.86 |
|  | African American | 40-54 | 0.72 | 0.37 | 1.39 |
|  | African American | 55-64 | 1.04 | 0.70 | 1.55 |
|  | African American | 65+ | 1.20 | 0.85 | 1.71 |
|  | Hispanic | 18-39 | 0.99 | 0.09 | 10.67 |
|  | Hispanic | 40-54 | 0.86 | 0.30 | 2.52 |
|  | Hispanic | 55-64 | 1.10 | 0.55 | 2.23 |
|  | Hispanic | 65+ | 1.03 | 0.64 | 1.66 |
| Current Smoker | Caucasian | 18-39 | 1.02 | 0.67 | 1.55 |
| (Current vs. Never) | Caucasian | 40-54 | 0.63 | 0.45 | 0.88 |
|  | Caucasian | 55-64 | 0.98 | 0.80 | 1.20 |
|  | Caucasian | 65+ | 1.13 | 0.92 | 1.39 |
|  | African American | 18-39 | 1.83 | 0.74 | 4.51 |
|  | African American | 40-54 | 2.56 | 1.00 | 6.58 |
|  | African American | 55-64 | 1.16 | 0.74 | 1.81 |
|  | African American | 65+ | 0.83 | 0.58 | 1.20 |
|  | Hispanic | 18-39 |  |  |  |
|  | Hispanic | 40-54 | 0.84 | 0.25 | 2.77 |
|  | Hispanic | 55-64 | 0.96 | 0.47 | 1.97 |
|  | Hispanic | 65+ | 0.87 | 0.53 | 1.42 |
| Current Smoker | Caucasian | 18-39 | 1.02 | 0.68 | 1.55 |
| (Former vs. Never) | Caucasian | 40-54 | 0.70 | 0.49 | 1.00 |
|  | Caucasian | 55-64 | 0.92 | 0.70 | 1.21 |
|  | Caucasian | 65+ | 1.76 | 1.25 | 2.49 |
|  | African American | 18-39 | 1.16 | 0.43 | 3.12 |
|  | African American | 40-54 | 2.85 | 1.02 | 7.95 |
|  | African American | 55-64 | 0.90 | 0.46 | 1.77 |
|  | African American | 65+ | 0.75 | 0.34 | 1.64 |
|  | Hispanic | 18-39 | 2.16 | 0.19 | 24.73 |
|  | Hispanic | 40-54 | 0.91 | 0.20 | 4.19 |
|  | Hispanic | 55-64 | 0.58 | 0.20 | 1.65 |
|  | Hispanic | 65+ | 0.80 | 0.34 | 1.92 |
| Household Income (& of Poverty Level) | Caucasian | 18-39 | 1.52 | 0.88 | 2.61 |
| 100-150% vs. 150%+ | Caucasian | 40-54 | 1.02 | 0.52 | 1.98 |
|  | Caucasian | 55-64 | 1.44 | 0.89 | 2.36 |
|  | Caucasian | 65+ | 2.07 | 1.23 | 3.51 |
|  | African American | 18-39 | 1.59 | 0.72 | 3.50 |
|  | African American | 40-54 | 1.48 | 0.67 | 3.30 |
|  | African American | 55-64 | 1.76 | 0.97 | 3.21 |
|  | African American | 65+ | 1.39 | 0.80 | 2.39 |
|  | Hispanic | 18-39 | 6.23 | 0.48 | 80.75 |
|  | Hispanic | 40-54 | 0.97 | 0.26 | 3.58 |
|  | Hispanic | 55-64 | 0.65 | 0.25 | 1.66 |
|  | Hispanic | 65+ | 0.99 | 0.49 | 2.00 |
| Household Income (& of Poverty Level) | Caucasian | 18-39 | 1.70 | 0.83 | 3.48 |
| <100% vs. 150%+ | Caucasian | 40-54 | 1.56 | 0.77 | 3.14 |
|  | Caucasian | 55-64 | 1.36 | 0.79 | 2.35 |
|  | Caucasian | 65+ | 2.59 | 1.37 | 4.87 |
|  | African American | 18-39 | 1.59 | 0.68 | 3.71 |
|  | African American | 40-54 | 1.99 | 0.79 | 4.98 |
|  | African American | 55-64 | 1.98 | 1.10 | 3.57 |
|  | African American | 65+ | 2.66 | 1.49 | 4.76 |
|  | Hispanic | 18-39 | 2.58 | 0.14 | 46.72 |
|  | Hispanic | 40-54 | 0.50 | 0.11 | 2.18 |
|  | Hispanic | 55-64 | 1.36 | 0.56 | 3.28 |
|  | Hispanic | 65+ | 0.79 | 0.42 | 1.51 |
| Education | Caucasian | 18-39 | 0.84 | 0.49 | 1.43 |
| High School Grade vs. Less than High School | Caucasian | 40-54 | 1.03 | 0.52 | 2.04 |
|  | Caucasian | 55-64 | 0.68 | 0.39 | 1.19 |
|  | Caucasian | 65+ | 0.73 | 0.45 | 1.21 |
|  | African American | 18-39 | 0.53 | 0.25 | 1.16 |
|  | African American | 40-54 | 0.81 | 0.36 | 1.83 |
|  | African American | 55-64 | 0.95 | 0.50 | 1.82 |
|  | African American | 65+ | 0.83 | 0.44 | 1.54 |
|  | Hispanic | 18-39 |  |  |  |
|  | Hispanic | 40-54 | 1.04 | 0.27 | 3.98 |
|  | Hispanic | 55-64 | 0.37 | 0.14 | 1.01 |
|  | Hispanic | 65+ | 1.11 | 0.59 | 2.07 |
| Education | Caucasian | 18-39 | 1.26 | 0.78 | 2.04 |
| Some College vs Less than High School | Caucasian | 40-54 | 2.16 | 1.14 | 4.07 |
|  | Caucasian | 55-64 | 1.09 | 0.63 | 1.87 |
|  | Caucasian | 65+ | 0.85 | 0.53 | 1.36 |
|  | African American | 18-39 | 0.52 | 0.22 | 1.23 |
|  | African American | 40-54 | 1.10 | 0.48 | 2.53 |
|  | African American | 55-64 | 1.09 | 0.57 | 2.06 |
|  | African American | 65+ | 0.71 | 0.38 | 1.30 |
|  | Hispanic | 18-39 | 3.58 | 0.42 | 30.42 |
|  | Hispanic | 40-54 | 4.36 | 1.15 | 16.44 |
|  | Hispanic | 55-64 | 1.11 | 0.50 | 2.46 |
|  | Hispanic | 65+ | 1.42 | 0.77 | 2.62 |
| Overall Health | Caucasian | 18-39 | 1.63 | 0.98 | 2.71 |
| (Good vs. Excellent) | Caucasian | 40-54 | 0.91 | 0.67 | 1.24 |
|  | Caucasian | 55-64 | 1.45 | 1.18 | 1.77 |
|  | Caucasian | 65+ | 1.54 | 1.26 | 1.88 |
|  | African American | 18-39 | 0.56 | 0.16 | 2.04 |
|  | African American | 40-54 | 1.03 | 0.38 | 2.78 |
|  | African American | 55-64 | 1.32 | 0.75 | 2.34 |
|  | African American | 65+ | 0.97 | 0.67 | 1.42 |
|  | Hispanic | 18-39 | 0.77 | 0.04 | 14.05 |
|  | Hispanic | 40-54 | 1.63 | 0.27 | 9.78 |
|  | Hispanic | 55-64 | 1.14 | 0.48 | 2.71 |
|  | Hispanic | 65+ | 0.89 | 0.52 | 1.52 |
| Overall Health | Caucasian | 18-39 | 2.63 | 1.56 | 4.44 |
| (Poor vs. Excellent) | Caucasian | 40-54 | 1.27 | 0.89 | 1.83 |
|  | Caucasian | 55-64 | 2.95 | 2.20 | 3.95 |
|  | Caucasian | 65+ | 3.23 | 2.25 | 4.63 |
|  | African American | 18-39 | 2.20 | 0.73 | 6.63 |
|  | African American | 40-54 | 1.68 | 0.63 | 4.52 |
|  | African American | 55-64 | 3.46 | 1.93 | 6.20 |
|  | African American | 65+ | 2.12 | 1.22 | 3.71 |
|  | Hispanic | 18-39 | 0.88 | 0.07 | 11.03 |
|  | Hispanic | 40-54 | 3.98 | 0.73 | 21.66 |
|  | Hispanic | 55-64 | 2.17 | 0.92 | 5.12 |
|  | Hispanic | 65+ | 1.65 | 0.86 | 3.19 |
