# Supplementary material for: What stresses men? predictors of perceived stress in a population-based multi-ethnic cross sectional cohort
Source: BMC Public Health. 2013 Feb 6;13:113. doi: 10.1186/1471-2458-13-113 (PMC3627635; doi:10.1186/1471-2458-13-113)
Supplement: Additional file 3: Table S3 — OR Estimates with Lower and Upper Bound of the 95% C. [file 1471-2458-13-113-S3.doc]

**Supplementary Table 3: OR Estimates with Lower and Upper Bound of the 95% CI**

| **Figure** | **Risk Factor** | **Race** | **Risk Factor Level** | **Age Group** | **OR** | **95%CI Lower Bound** | **95%CI Upper Bound** |
| --- | --- | --- | --- | --- | --- | --- | --- |
| 1 | Uninsured | Caucasian | 0 | 65+ | (REF) |  |  |
|  |  | Caucasian | 0 | 55-64 | 2.70 | 2.16 | 3.37 |
|  |  | Caucasian | 0 | 40-54 | 4.88 | 4.01 | 5.95 |
|  |  | Caucasian | 0 | 18-39 | 5.55 | 4.52 | 6.82 |
|  |  | Caucasian | 1 | 65+ | 6.18 | 3.17 | 12.05 |
|  |  | Caucasian | 1 | 55-64 | 3.44 | 2.05 | 5.77 |
|  |  | Caucasian | 1 | 40-54 | 7.28 | 5.27 | 10.07 |
|  |  | Caucasian | 1 | 18-39 | 7.47 | 5.65 | 9.87 |
|  |  | African American | 0 | 65+ | 0.87 | 0.58 | 1.31 |
|  |  | African American | 0 | 55-64 | 1.98 | 1.35 | 2.89 |
|  |  | African American | 0 | 40-54 | 3.32 | 2.52 | 4.39 |
|  |  | African American | 0 | 18-39 | 4.84 | 3.67 | 6.37 |
|  |  | African American | 1 | 65+ | 4.23 | 1.79 | 10.00 |
|  |  | African American | 1 | 55-64 | 2.56 | 1.28 | 5.11 |
|  |  | African American | 1 | 40-54 | 3.92 | 2.51 | 6.14 |
|  |  | African American | 1 | 18-39 | 5.87 | 4.15 | 8.29 |
|  |  | Hispanic | 0 | 65+ | 0.56 | 0.17 | 1.86 |
|  |  | Hispanic | 0 | 55-64 | 2.80 | 1.43 | 5.48 |
|  |  | Hispanic | 0 | 40-54 | 3.20 | 2.07 | 4.93 |
|  |  | Hispanic | 0 | 18-39 | 5.33 | 3.74 | 7.61 |
|  |  | Hispanic | 1 | 65+ | 2.18 | 0.23 | 20.45 |
|  |  | Hispanic | 1 | 55-64 | 7.29 | 2.73 | 19.50 |
|  |  | Hispanic | 1 | 40-54 | 3.07 | 1.65 | 5.72 |
|  |  | Hispanic | 1 | 18-39 | 3.90 | 2.62 | 5.82 |

| **Figure** | **Risk Factor** | **Race** | **Risk Factor Level** | **Age Group** | **OR** | **95%CI Lower Bound** | **95%CI Upper Bound** |
| --- | --- | --- | --- | --- | --- | --- | --- |
| 1 | Cut A Meal | Caucasian | 0 | 65+ | (REF) |  |  |
|  |  | Caucasian | 0 | 55-64 | 2.53 | 2.03 | 3.15 |
|  |  | Caucasian | 0 | 40-54 | 4.42 | 3.64 | 5.38 |
|  |  | Caucasian | 0 | 18-39 | 5.04 | 4.13 | 6.15 |
|  |  | Caucasian | 1 | 65+ | 6.91 | 3.10 | 15.43 |
|  |  | Caucasian | 1 | 55-64 | 6.38 | 3.51 | 11.57 |
|  |  | Caucasian | 1 | 40-54 | 20.57 | 13.35 | 31.69 |
|  |  | Caucasian | 1 | 18-39 | 19.98 | 13.22 | 30.18 |
|  |  | African American | 0 | 65+ | 0.87 | 0.58 | 1.30 |
|  |  | African American | 0 | 55-64 | 2.11 | 1.48 | 3.02 |
|  |  | African American | 0 | 40-54 | 3.17 | 2.41 | 4.15 |
|  |  | African American | 0 | 18-39 | 4.40 | 3.40 | 5.69 |
|  |  | African American | 1 | 65+ | 23.36 | 6.24 | 87.45 |
|  |  | African American | 1 | 55-64 | 2.27 | 0.80 | 6.46 |
|  |  | African American | 1 | 40-54 | 5.50 | 3.28 | 9.21 |
|  |  | African American | 1 | 18-39 | 10.15 | 6.36 | 16.19 |
|  |  | Hispanic | 0 | 65+ | 0.83 | 0.29 | 2.39 |
|  |  | Hispanic | 0 | 55-64 | 3.08 | 1.66 | 5.69 |
|  |  | Hispanic | 0 | 40-54 | 2.53 | 1.66 | 3.86 |
|  |  | Hispanic | 0 | 18-39 | 4.41 | 3.25 | 6.00 |
|  |  | Hispanic | 1 | 65+ | NE |  |  |
|  |  | Hispanic | 1 | 55-64 | 11.86 | 3.27 | 43.10 |
|  |  | Hispanic | 1 | 40-54 | 8.01 | 3.81 | 16.85 |
|  |  | Hispanic | 1 | 18-39 | 6.61 | 3.53 | 12.36 |

| **Figure** | **Risk Factor** | **Race** | **Risk Factor Level** | **Age Group** | **OR** | **95%CI Lower Bound** | **95%CI Upper Bound** |
| --- | --- | --- | --- | --- | --- | --- | --- |
| 1 | Prescription not Filled | Caucasian | 0 | 65+ | (REF) |  |  |
|  |  | Caucasian | 0 | 55-64 | 2.39 | 1.91 | 2.99 |
|  |  | Caucasian | 0 | 40-54 | 4.44 | 3.64 | 5.42 |
|  |  | Caucasian | 0 | 18-39 | 5.04 | 4.11 | 6.17 |
|  |  | Caucasian | 1 | 65+ | 2.19 | 1.29 | 3.75 |
|  |  | Caucasian | 1 | 55-64 | 7.36 | 4.65 | 11.64 |
|  |  | Caucasian | 1 | 40-54 | 10.74 | 7.69 | 14.98 |
|  |  | Caucasian | 1 | 18-39 | 13.53 | 9.54 | 19.19 |
|  |  | African American | 0 | 65+ | 0.89 | 0.59 | 1.35 |
|  |  | African American | 0 | 55-64 | 1.86 | 1.27 | 2.71 |
|  |  | African American | 0 | 40-54 | 2.96 | 2.23 | 3.92 |
|  |  | African American | 0 | 18-39 | 4.53 | 3.50 | 5.87 |
|  |  | African American | 1 | 65+ | 2.44 | 1.20 | 4.95 |
|  |  | African American | 1 | 55-64 | 3.49 | 1.69 | 7.22 |
|  |  | African American | 1 | 40-54 | 5.52 | 3.54 | 8.60 |
|  |  | African American | 1 | 18-39 | 7.92 | 4.95 | 12.67 |
|  |  | Hispanic | 0 | 65+ | 0.59 | 0.18 | 1.97 |
|  |  | Hispanic | 0 | 55-64 | 3.34 | 1.76 | 6.33 |
|  |  | Hispanic | 0 | 40-54 | 2.75 | 1.81 | 4.18 |
|  |  | Hispanic | 0 | 18-39 | 3.59 | 2.62 | 4.92 |
|  |  | Hispanic | 1 | 65+ | 1.54 | 0.17 | 14.17 |
|  |  | Hispanic | 1 | 55-64 | 5.04 | 1.82 | 13.93 |
|  |  | Hispanic | 1 | 40-54 | 4.90 | 2.40 | 10.02 |
|  |  | Hispanic | 1 | 18-39 | 14.88 | 7.89 | 28.06 |

| **Figure** | **Risk Factor** | **Race** | **Risk Factor Level** | **Age Group** | **OR** | **95%CI Lower Bound** | **95%CI Upper Bound** |
| --- | --- | --- | --- | --- | --- | --- | --- |
| 2 | Chronic Condition | Caucasian | 0 | 65+ | (REF) |  |  |
|  |  | Caucasian | 0 | 55-64 | 2.62 | 1.98 | 3.46 |
|  |  | Caucasian | 0 | 40-54 | 4.18 | 3.27 | 5.35 |
|  |  | Caucasian | 0 | 18-39 | 5.10 | 4.00 | 6.51 |
|  |  | Caucasian | 1 | 65+ | 1.06 | 0.77 | 1.47 |
|  |  | Caucasian | 1 | 55-64 | 2.55 | 1.86 | 3.49 |
|  |  | Caucasian | 1 | 40-54 | 6.57 | 4.95 | 8.72 |
|  |  | Caucasian | 1 | 18-39 | 8.66 | 5.99 | 12.52 |
|  |  | African American | 0 | 65+ | 0.76 | 0.43 | 1.34 |
|  |  | African American | 0 | 55-64 | 1.79 | 1.12 | 2.85 |
|  |  | African American | 0 | 40-54 | 2.59 | 1.87 | 3.60 |
|  |  | African American | 0 | 18-39 | 4.74 | 3.56 | 6.32 |
|  |  | African American | 1 | 65+ | 1.25 | 0.77 | 2.01 |
|  |  | African American | 1 | 55-64 | 2.18 | 1.32 | 3.62 |
|  |  | African American | 1 | 40-54 | 5.00 | 3.31 | 7.56 |
|  |  | African American | 1 | 18-39 | 4.91 | 2.76 | 8.74 |
|  |  | Hispanic | 0 | 65+ | 0.83 | 0.19 | 3.62 |
|  |  | Hispanic | 0 | 55-64 | 3.23 | 1.48 | 7.05 |
|  |  | Hispanic | 0 | 40-54 | 2.36 | 1.47 | 3.79 |
|  |  | Hispanic | 0 | 18-39 | 3.96 | 2.83 | 5.55 |
|  |  | Hispanic | 1 | 65+ | 0.53 | 0.12 | 2.31 |
|  |  | Hispanic | 1 | 55-64 | 3.72 | 1.72 | 8.06 |
|  |  | Hispanic | 1 | 40-54 | 4.55 | 2.47 | 8.36 |
|  |  | Hispanic | 1 | 18-39 | 6.61 | 3.41 | 12.81 |

| **Figure** | **Risk Factor** | **Race** | **Risk Factor Level** | **Age Group** | **OR** | **95%CI Lower Bound** | **95%CI Upper Bound** |
| --- | --- | --- | --- | --- | --- | --- | --- |
| 2 | Mental Health Condition | Caucasian | 0 | 65+ | (REF) |  |  |
|  |  | Caucasian | 0 | 55-64 | 2.46 | 1.95 | 3.10 |
|  |  | Caucasian | 0 | 40-54 | 4.58 | 3.74 | 5.62 |
|  |  | Caucasian | 0 | 18-39 | 5.25 | 4.26 | 6.45 |
|  |  | Caucasian | 1 | 65+ | 3.05 | 1.89 | 4.93 |
|  |  | Caucasian | 1 | 55-64 | 6.17 | 4.18 | 9.11 |
|  |  | Caucasian | 1 | 40-54 | 10.41 | 7.64 | 14.18 |
|  |  | Caucasian | 1 | 18-39 | 13.62 | 9.77 | 18.99 |
|  |  | African American | 0 | 65+ | 1.10 | 0.75 | 1.60 |
|  |  | African American | 0 | 55-64 | 1.74 | 1.19 | 2.55 |
|  |  | African American | 0 | 40-54 | 3.15 | 2.39 | 4.16 |
|  |  | African American | 0 | 18-39 | 4.69 | 3.64 | 6.06 |
|  |  | African American | 1 | 65+ | 2.23 | 0.58 | 8.58 |
|  |  | African American | 1 | 55-64 | 9.27 | 3.63 | 23.72 |
|  |  | African American | 1 | 40-54 | 6.23 | 3.75 | 10.36 |
|  |  | African American | 1 | 18-39 | 15.96 | 7.62 | 33.42 |
|  |  | Hispanic | 0 | 65+ | 0.66 | 0.20 | 2.19 |
|  |  | Hispanic | 0 | 55-64 | 3.23 | 1.74 | 5.98 |
|  |  | Hispanic | 0 | 40-54 | 2.78 | 1.84 | 4.21 |
|  |  | Hispanic | 0 | 18-39 | 4.03 | 2.96 | 5.51 |
|  |  | Hispanic | 1 | 65+ | 1.29 | 0.15 | 11.27 |
|  |  | Hispanic | 1 | 55-64 | 8.47 | 2.19 | 32.75 |
|  |  | Hispanic | 1 | 40-54 | 6.76 | 3.10 | 14.77 |
|  |  | Hispanic | 1 | 18-39 | 17.32 | 8.12 | 36.98 |

| **Figure** | **Risk Factor** | **Race** | **Risk Factor Level** | **Age Group** | **OR** | **95%CI Lower Bound** | **95%CI Upper Bound** |
| --- | --- | --- | --- | --- | --- | --- | --- |
| 2 | Hospitalized | Caucasian | 0 | 65+ | (REF) |  |  |
|  |  | Caucasian | 0 | 55-64 | 2.75 | 2.16 | 3.51 |
|  |  | Caucasian | 0 | 40-54 | 4.68 | 3.77 | 5.80 |
|  |  | Caucasian | 0 | 18-39 | 5.65 | 4.55 | 7.03 |
|  |  | Caucasian | 1 | 65+ | 1.59 | 1.11 | 2.29 |
|  |  | Caucasian | 1 | 55-64 | 3.18 | 2.17 | 4.67 |
|  |  | Caucasian | 1 | 40-54 | 12.46 | 8.84 | 17.58 |
|  |  | Caucasian | 1 | 18-39 | 11.62 | 7.52 | 17.97 |
|  |  | African American | 0 | 65+ | 0.89 | 0.56 | 1.40 |
|  |  | African American | 0 | 55-64 | 1.97 | 1.32 | 2.93 |
|  |  | African American | 0 | 40-54 | 3.60 | 2.71 | 4.79 |
|  |  | African American | 0 | 18-39 | 4.81 | 3.68 | 6.29 |
|  |  | African American | 1 | 65+ | 1.77 | 0.99 | 3.16 |
|  |  | African American | 1 | 55-64 | 2.87 | 1.49 | 5.52 |
|  |  | African American | 1 | 40-54 | 3.07 | 1.82 | 5.19 |
|  |  | African American | 1 | 18-39 | 9.20 | 5.27 | 16.06 |
|  |  | Hispanic | 0 | 65+ | 0.49 | 0.11 | 2.09 |
|  |  | Hispanic | 0 | 55-64 | 3.41 | 1.79 | 6.48 |
|  |  | Hispanic | 0 | 40-54 | 2.86 | 1.89 | 4.34 |
|  |  | Hispanic | 0 | 18-39 | 4.32 | 3.15 | 5.92 |
|  |  | Hispanic | 1 | 65+ | 1.34 | 0.28 | 6.42 |
|  |  | Hispanic | 1 | 55-64 | 3.91 | 1.22 | 12.51 |
|  |  | Hispanic | 1 | 40-54 | 5.89 | 2.57 | 13.48 |
|  |  | Hispanic | 1 | 18-39 | 8.97 | 4.22 | 19.06 |

| **Figure** | **Risk Factor** | **Race** | **Risk Factor Level** | **Age Group** | **OR** | **95%CI Lower Bound** | **95%CI Upper Bound** |
| --- | --- | --- | --- | --- | --- | --- | --- |
| 3 | Subject to Physical Violence | Caucasian | 0 | 65+ | (REF) |  |  |
|  |  | Caucasian | 0 | 55-64 | 2.55 | 2.06 | 3.16 |
|  |  | Caucasian | 0 | 40-54 | 4.68 | 3.86 | 5.67 |
|  |  | Caucasian | 0 | 18-39 | 5.34 | 4.38 | 6.49 |
|  |  | Caucasian | 1 | 65+ | 4.83 | 1.67 | 13.96 |
|  |  | Caucasian | 1 | 55-64 | 6.61 | 2.86 | 15.24 |
|  |  | Caucasian | 1 | 40-54 | 12.52 | 7.46 | 21.01 |
|  |  | Caucasian | 1 | 18-39 | 9.76 | 6.63 | 14.37 |
|  |  | African American | 0 | 65+ | 0.96 | 0.66 | 1.40 |
|  |  | African American | 0 | 55-64 | 1.97 | 1.39 | 2.79 |
|  |  | African American | 0 | 40-54 | 3.04 | 2.34 | 3.95 |
|  |  | African American | 0 | 18-39 | 4.31 | 3.34 | 5.55 |
|  |  | African American | 1 | 65+ | 2.68 | 0.51 | 14.12 |
|  |  | African American | 1 | 55-64 | 1.42 | 0.16 | 12.43 |
|  |  | African American | 1 | 40-54 | 7.40 | 3.50 | 15.64 |
|  |  | African American | 1 | 18-39 | 10.67 | 6.39 | 17.81 |
|  |  | Hispanic | 0 | 65+ | 0.64 | 0.22 | 1.84 |
|  |  | Hispanic | 0 | 55-64 | 3.32 | 1.88 | 5.87 |
|  |  | Hispanic | 0 | 40-54 | 2.93 | 2.00 | 4.28 |
|  |  | Hispanic | 0 | 18-39 | 3.71 | 2.73 | 5.05 |
|  |  | Hispanic | 1 | 65+ | NE |  |  |
|  |  | Hispanic | 1 | 55-64 | 8.08 | 0.72 | 90.97 |
|  |  | Hispanic | 1 | 40-54 | 4.93 | 1.07 | 22.67 |
|  |  | Hispanic | 1 | 18-39 | 11.39 | 6.02 | 21.54 |

| **Figure** | **Risk Factor** | **Race** | **Risk Factor Level** | **Age Group** | **OR** | **95%CI Lower Bound** | **95%CI Upper Bound** |
| --- | --- | --- | --- | --- | --- | --- | --- |
| 3 | Lives Alone | Caucasian | 0 | 65+ | (REF) |  |  |
|  |  | Caucasian | 0 | 55-64 | 2.64 | 2.05 | 3.42 |
|  |  | Caucasian | 0 | 40-54 | 4.86 | 3.86 | 6.12 |
|  |  | Caucasian | 0 | 18-39 | 5.92 | 4.69 | 7.46 |
|  |  | Caucasian | 1 | 65+ | 1.11 | 0.79 | 1.55 |
|  |  | Caucasian | 1 | 55-64 | 2.69 | 1.91 | 3.79 |
|  |  | Caucasian | 1 | 40-54 | 5.21 | 3.92 | 6.91 |
|  |  | Caucasian | 1 | 18-39 | 4.99 | 3.69 | 6.74 |
|  |  | African American | 0 | 65+ | 1.16 | 0.72 | 1.86 |
|  |  | African American | 0 | 55-64 | 2.11 | 1.38 | 3.24 |
|  |  | African American | 0 | 40-54 | 3.47 | 2.55 | 4.72 |
|  |  | African American | 0 | 18-39 | 4.90 | 3.68 | 6.51 |
|  |  | African American | 1 | 65+ | 0.81 | 0.47 | 1.41 |
|  |  | African American | 1 | 55-64 | 1.70 | 0.98 | 2.97 |
|  |  | African American | 1 | 40-54 | 2.88 | 1.91 | 4.35 |
|  |  | African American | 1 | 18-39 | 4.92 | 3.21 | 7.55 |
|  |  | Hispanic | 0 | 65+ | 0.48 | 0.11 | 2.06 |
|  |  | Hispanic | 0 | 55-64 | 3.49 | 1.84 | 6.65 |
|  |  | Hispanic | 0 | 40-54 | 2.94 | 1.91 | 4.52 |
|  |  | Hispanic | 0 | 18-39 | 4.16 | 2.99 | 5.78 |
|  |  | Hispanic | 1 | 65+ | 0.94 | 0.20 | 4.35 |
|  |  | Hispanic | 1 | 55-64 | 3.35 | 1.16 | 9.64 |
|  |  | Hispanic | 1 | 40-54 | 3.06 | 1.47 | 6.39 |
|  |  | Hispanic | 1 | 18-39 | 6.08 | 3.23 | 11.47 |

| **Figure** | **Risk Factor** | **Race** | **Risk Factor Level** | **Age Group** | **OR** | **95%CI Lower Bound** | **95%CI Upper Bound** |
| --- | --- | --- | --- | --- | --- | --- | --- |
| 3 | Firearms in the Home | Caucasian | 0 | 65+ | (REF) |  |  |
|  |  | Caucasian | 0 | 55-64 | 2.55 | 1.98 | 3.29 |
|  |  | Caucasian | 0 | 40-54 | 5.05 | 4.04 | 6.30 |
|  |  | Caucasian | 0 | 18-39 | 5.44 | 4.34 | 6.82 |
|  |  | Caucasian | 1 | 65+ | 1.09 | 0.76 | 1.58 |
|  |  | Caucasian | 1 | 55-64 | 3.05 | 2.23 | 4.18 |
|  |  | Caucasian | 1 | 40-54 | 4.90 | 3.75 | 6.40 |
|  |  | Caucasian | 1 | 18-39 | 6.96 | 5.26 | 9.21 |
|  |  | African American | 0 | 65+ | 1.02 | 0.67 | 1.55 |
|  |  | African American | 0 | 55-64 | 2.06 | 1.38 | 3.06 |
|  |  | African American | 0 | 40-54 | 3.22 | 2.41 | 4.30 |
|  |  | African American | 0 | 18-39 | 4.67 | 3.54 | 6.16 |
|  |  | African American | 1 | 65+ | 0.83 | 0.37 | 1.87 |
|  |  | African American | 1 | 55-64 | 1.72 | 0.87 | 3.43 |
|  |  | African American | 1 | 40-54 | 3.53 | 2.07 | 6.03 |
|  |  | African American | 1 | 18-39 | 6.00 | 3.82 | 9.43 |
|  |  | Hispanic | 0 | 65+ | 0.83 | 0.28 | 2.42 |
|  |  | Hispanic | 0 | 55-64 | 3.01 | 1.59 | 5.71 |
|  |  | Hispanic | 0 | 40-54 | 3.04 | 2.01 | 4.60 |
|  |  | Hispanic | 0 | 18-39 | 4.25 | 3.08 | 5.86 |
|  |  | Hispanic | 1 | 65+ | NE |  |  |
|  |  | Hispanic | 1 | 55-64 | 4.14 | 1.23 | 13.91 |
|  |  | Hispanic | 1 | 40-54 | 3.28 | 1.41 | 7.61 |
|  |  | Hispanic | 1 | 18-39 | 7.30 | 3.66 | 14.54 |

| **Figure** | **Risk Factor** | **Race** | **Risk Factor Level** | **Age Group** | **OR** | **95%CI Lower Bound** | **95%CI Upper Bound** |
| --- | --- | --- | --- | --- | --- | --- | --- |
| 4 | Exercise | Caucasian | 0 | 65+ | (REF) |  |  |
|  |  | Caucasian | 0 | 55-64 | 2.97 | 2.26 | 3.89 |
|  |  | Caucasian | 0 | 40-54 | 4.95 | 3.88 | 6.31 |
|  |  | Caucasian | 0 | 18-39 | 5.99 | 4.67 | 7.69 |
|  |  | Caucasian | 1 | 65+ | 1.23 | 0.89 | 1.70 |
|  |  | Caucasian | 1 | 55-64 | 2.37 | 1.72 | 3.26 |
|  |  | Caucasian | 1 | 40-54 | 5.39 | 4.13 | 7.03 |
|  |  | Caucasian | 1 | 18-39 | 5.71 | 4.38 | 7.46 |
|  |  | African American | 0 | 65+ | 1.35 | 0.88 | 2.08 |
|  |  | African American | 0 | 55-64 | 2.23 | 1.45 | 3.42 |
|  |  | African American | 0 | 40-54 | 3.30 | 2.36 | 4.62 |
|  |  | African American | 0 | 18-39 | 4.72 | 3.44 | 6.46 |
|  |  | African American | 1 | 65+ | 0.51 | 0.24 | 1.09 |
|  |  | African American | 1 | 55-64 | 1.61 | 0.89 | 2.88 |
|  |  | African American | 1 | 40-54 | 3.45 | 2.40 | 4.98 |
|  |  | African American | 1 | 18-39 | 5.68 | 4.02 | 8.03 |
|  |  | Hispanic | 0 | 65+ | 0.66 | 0.20 | 2.26 |
|  |  | Hispanic | 0 | 55-64 | 3.92 | 1.91 | 8.05 |
|  |  | Hispanic | 0 | 40-54 | 3.00 | 1.89 | 4.76 |
|  |  | Hispanic | 0 | 18-39 | 4.54 | 3.13 | 6.60 |
|  |  | Hispanic | 1 | 65+ | 0.65 | 0.08 | 5.17 |
|  |  | Hispanic | 1 | 55-64 | 3.38 | 1.44 | 7.93 |
|  |  | Hispanic | 1 | 40-54 | 3.31 | 1.80 | 6.10 |
|  |  | Hispanic | 1 | 18-39 | 4.66 | 3.03 | 7.16 |

| **Figure** | **Risk Factor** | **Race** | **Risk Factor Level** | **Age Group** | **OR** | **95%CI Lower Bound** | **95%CI Upper Bound** |
| --- | --- | --- | --- | --- | --- | --- | --- |
| 4 | Current Smoker | Caucasian | 0 | 65+ | (REF) |  |  |
|  |  | Caucasian | 0 | 55-64 | 3.49 | 2.32 | 5.25 |
|  |  | Caucasian | 0 | 40-54 | 4.95 | 3.45 | 7.11 |
|  |  | Caucasian | 0 | 18-39 | 4.92 | 3.41 | 7.10 |
|  |  | Caucasian | 1 | 65+ | 1.02 | 0.67 | 1.55 |
|  |  | Caucasian | 1 | 55-64 | 2.20 | 1.49 | 3.24 |
|  |  | Caucasian | 1 | 40-54 | 4.83 | 3.41 | 6.85 |
|  |  | Caucasian | 1 | 18-39 | 5.56 | 3.92 | 7.88 |
|  |  | Caucasian | 2 | 65+ | 1.02 | 0.68 | 1.55 |
|  |  | Caucasian | 2 | 55-64 | 2.45 | 1.63 | 3.70 |
|  |  | Caucasian | 2 | 40-54 | 4.55 | 3.07 | 6.75 |
|  |  | Caucasian | 2 | 18-39 | 8.66 | 5.55 | 13.52 |
|  |  | African American | 0 | 65+ | 0.68 | 0.29 | 1.60 |
|  |  | African American | 0 | 55-64 | 0.86 | 0.34 | 2.14 |
|  |  | African American | 0 | 40-54 | 2.96 | 1.81 | 4.84 |
|  |  | African American | 0 | 18-39 | 5.38 | 3.49 | 8.28 |
|  |  | African American | 1 | 65+ | 1.25 | 0.72 | 2.18 |
|  |  | African American | 1 | 55-64 | 2.20 | 1.31 | 3.70 |
|  |  | African American | 1 | 40-54 | 3.42 | 2.27 | 5.16 |
|  |  | African American | 1 | 18-39 | 4.48 | 3.00 | 6.69 |
|  |  | African American | 2 | 65+ | 0.79 | 0.40 | 1.58 |
|  |  | African American | 2 | 55-64 | 2.44 | 1.27 | 4.71 |
|  |  | African American | 2 | 40-54 | 2.67 | 1.39 | 5.12 |
|  |  | African American | 2 | 18-39 | 4.03 | 1.81 | 8.96 |
|  |  | Hispanic | 0 | 65+ | 0.65 | 0.08 | 5.21 |
|  |  | Hispanic | 0 | 55-64 | 3.77 | 1.39 | 10.22 |
|  |  | Hispanic | 0 | 40-54 | 3.21 | 1.75 | 5.90 |
|  |  | Hispanic | 0 | 18-39 | 4.72 | 2.87 | 7.74 |
|  |  | Hispanic | 1 | 65+ | NE |  |  |
|  |  | Hispanic | 1 | 55-64 | 3.15 | 1.40 | 7.11 |
|  |  | Hispanic | 1 | 40-54 | 3.08 | 1.70 | 5.61 |
|  |  | Hispanic | 1 | 18-39 | 4.09 | 2.57 | 6.50 |
|  |  | Hispanic | 2 | 65+ | 1.41 | 0.36 | 5.46 |
|  |  | Hispanic | 2 | 55-64 | 3.42 | 0.98 | 11.95 |
|  |  | Hispanic | 2 | 40-54 | 1.86 | 0.71 | 4.89 |
|  |  | Hispanic | 2 | 18-39 | 3.79 | 1.62 | 8.88 |
